# Evidence of Slow Neural Processing, Developmental Differences and Sensitivity to Cannabis Effects in a Sample at Clinical High Risk for Psychosis From the NAPLS Consortium Assessed With the Human Startle Paradigm

**DOI:** 10.3389/fpsyt.2020.00833

**Published:** 2020-08-25

**Authors:** Kristin S. Cadenhead, Erica Duncan, Jean Addington, Carrie Bearden, Tyrone D. Cannon, Barbara A. Cornblatt, Dan Mathalon, Thomas H. McGlashan, Diana O. Perkins, Larry J. Seidman, Ming Tsuang, Elaine F. Walker, Scott W. Woods, Peter Bauchman, Ayse Belger, Ricardo E. Carrión, Franc Donkers, Jason Johannesen, Gregory Light, Margaret Niznikiewicz, Jason Nunag, Brian Roach

**Affiliations:** ^1^Department of Psychiatry, University of California San Diego (UCSD), La Jolla, CA, United States; ^2^Department of Psychiatry, Atlanta Veterans Affairs Healthcare System, Decatur, GA, United States; ^3^Department of Psychiatry, Emory University School of Medicine, Atlanta, GA, United States; ^4^Hotchkiss Brain Institute, University of Calgary, Calgary, AB, Canada; ^5^Department of Psychiatry and Psychology, University of California Los Angeles (UCLA), Los Angeles, CA, United States; ^6^Department of Psychiatry and Psychology, Yale University, New Haven, CT, United States; ^7^Department of Psychiatry and Psychology, The Feinstein Institute for Medical Research, Manhasset, NY, United States; ^8^Department of Psychology, Hofstra North Shore-LIJ School of Medicine, Hempstead, NY, United States; ^9^The Zucker Hillside Hospital, New York, NY, United States; ^10^University of California, San Francisco, San Francisco, CA, United States; ^11^San Francisco VA Medical Center, San Francisco, VA, United States; ^12^University of North Carolina (UNC), Chapel Hill, NC, United States; ^13^Department of Psychiatry, Harvard University, Boston, MA, United States

**Keywords:** prodrome, schizophrenia, cannabis, latency, startle, prepulse inhibition, neurodevelopment, age

## Abstract

**Abstract:**

Biomarkers are important in the study of the prodromal period of psychosis because they can help to identify individuals at greatest risk for future psychotic illness and provide insights into disease mechanism underlying neurodevelopmental abnormalities. The biomarker abnormalities can then be targeted with treatment, with an aim toward prevention or mitigation of disease. The human startle paradigm has been used in translational studies of psychopathology including psychotic illness to assess preattentive information processing for over 50 years. In one of the largest studies to date in clinical high risk (CHR) for psychosis participants, we aimed to evaluate startle indices as biomarkers of risk along with the role of age, sex, treatment, and substance use in this population of high risk individuals.

**Methods:**

Startle response reactivity, latency, and prepulse inhibition (PPI) were assessed in 543 CHR and 218 Normal Comparison (NC) participants between the ages of 12 and 35.

**Results:**

At 1 year follow-up, 58 CHR participants had converted to psychosis. CHR and NC groups did not differ across any of the startle measures but those CHR participants who later converted to psychosis had significantly slower startle latency than did those who did not convert to psychosis, and this effect was driven by female CHR participants. PPI was significantly associated with age in the CHR, but not the NC, participants with the greatest positive age correlations present in those CHR participants who later converted to psychosis, consistent with a prior report. Finally, there was a significant group by cannabis use interaction due to greater PPI in cannabis users and opposite PPI group effects in users (CHR>NC) and non-users (NC>CHR).

**Discussion:**

This is the first study to demonstrate a relationship of startle response latency to psychotic conversion in a CHR population. PPI is an important biomarker that may be sensitive to the neurodevelopmental abnormalities thought to be present in psychosis prone individuals and the effects of cannabis. The significant correlations with age in this sample as well as the finding of greater PPI in CHR cannabis users replicate findings from another large sample of CHR participants.

## Introduction

A better understanding of the early stages of psychosis along with biomarkers linked to psychosis risk can lead to better treatments that target the mechanism of disease and perhaps alter the course of illness. The human startle paradigm is a well-studied translational biomarker with potential utility to better understand early psychopathological processes in the development of psychosis. Relatively few studies have reported on startle reactivity or prepulse inhibition (PPI) in participants at clinical high risk (CHR) for psychosis (1–5). One of the first studies by Quednow et al. (4) found reduced PPI in CHR (N = 52), consistent with findings in unmedicated first-episode patients with schizophrenia (N = 18). PPI deficits were also observed by Ziermans (5) who reported stable PPI deficits in CHR participants (N = 44), as did De Koning (1) who found reduced PPI in a small sample of CHR participants (N = 14) compared to normal comparison (NC) participants. More recently, Togay et al. (2) reported that (N = 29) CHR participants had reduced PPI deficits relative to NC and familial high-risk participants. Cadenhead (3) did not find PPI deficits in CHR (N = 89) compared to NC but did find significant sex, medication, cannabis and tobacco effects on startle measures. A small sample of CHR participants (N = 6) who later converted to psychosis in the Cadenhead study had greater PPI than both NC and CHR participants who did not convert to psychosis by 2 year follow-up. Importantly, a significant positive correlation between PPI and age was found in CHR participants in the Cadenhead study that was not present in NC, revealing possible neurodevelopmental differences in the early psychosis population (3).

PPI is an index of sensorimotor gating and is used to understand brain disorders that are characterized by gating deficits such as schizophrenia (6). PPI is stable with repeated testing (7–9) and has good within and between site reliability (10). PPI and other startle indices such as latency and magnitude of the startle response are also heritable (11, 12), suggesting the utility of startle measures as candidate biomarkers to better understand psychosis risk (13–15). A forebrain/pontine circuit has been identified in animal models that modulates PPI (16, 17). Neurotransmitters including dopamine, serotonin, and glutamate are central to this modulatory circuitry, and it is possible to use a variety of pharmacologic interventions (eg, amphetamine, apomorphine, phencyclidine, or ketamine) to disrupt PPI (18). Developmental models such as isolation rearing used to understand neurodevelopmental disorders such as schizophrenia can induce PPI deficits (19). Similarly, chronic pubertal cannabinoid administration during development in rats can also induce PPI deficits in adult animals after maturation (20) an interesting model for the possible epigenetic effects of cannabis in vulnerable CHR individuals.

The human startle paradigm was included in the second phase of the North American Prodrome Longitudinal Studies (NAPLS2) consortium to evaluate startle reactivity, latency, and PPI in participants at CHR for psychosis (N = 543) and non-psychiatric comparison participants (NC; N = 218). Given the mixed findings in previous CHR studies that could be accounted for by variables such as sex, age, drug or medication effects, another aim was to investigate group differences while further examining the role of these variables on startle measures (3). Finally, since the utility of startle measures in predicting risk for conversion to psychosis has not been assessed in an adequately powered sample, a final aim was to determine whether startle measures have utility as biomarkers of psychosis risk.

## Methods

### Participants

Startle magnitude (reactivity), latency, and PPI were assessed in 543 individuals at CHR for psychosis and 218 NC participants between the ages of 12 and 35 who participated in the NAPLS2 study (21). All eight sites used similar recruitment methods, and all participants were discussed as part of weekly consensus meetings to assure reliability across sites. All eight NAPLS sites received approval by institutional review boards. Written informed consent was obtained from all adult participants and parents/guardians of minors.

### Assessment

The Structured Interview for Psychosis-Risk Syndromes (SIPS) (22) and Structured Interview for DSM-IV Axis I were used to establish diagnostic criteria. The Alcohol and Drug Use Scale (AUS/DUS) (23) was used to assess history of drug or tobacco use.

CHR participants all fulfilled the Criteria of Psychosis-Risk Syndromes (COPS) from the Structured Interview for Psychosis-Risk Syndromes (SIPS) (22). NC did not meet criteria for DSM-IV Axis I, meet CHR criteria or report a family history of psychosis. Exclusion criteria included hearing impairment, serious head injury, or current substance misuse.

### Human Acoustic Startle Paradigm

Identical procedures and equipment were used at the nine NAPLS sites. Procedure manuals were developed and implemented across sites (10).

All research participants receive screening for hearing impairment (>45 dB 1000 Hz) prior to startle testing. Smoking was allowed up to 30 min prior to testing on the startle paradigm to avoid nicotine intoxication or withdrawal effects. We used a startle-stimulus generating program (Grace Design Model m902 Amplifier and Neurobehavioral Systems Presentation software) developed by UCSD at each site. We calibrated the sound at all sites with Quest 210 Sound Level Meters and assured 70 dB background noise and 115 dB for extended length startle bursts by adjusting the Amplifier. All sites used the same Biosemi systems (Biosemi, Amsterdam, Netherlands). Startle response was measured with EMG recording by electrodes (Ag/AgCl) placed at the outer canthus and below the right eye assuring resistances less than 10 kΩ (3). Headphones (TDH-39P) were used to present the startle stimuli binaurally. A background noise of 70 dB [A] and startle pulses (115 dB [A], 40 ms noise burst) were presented either alone or following a prepulse (86 dB [A], 20 ms noise burst) presented at interstimulus intervals (ISI) of 30, 60, or 120 ms. The paradigm includes a 5-min acclimation followed by five pulse alone stimuli (Block 1) then 30 trials that included six trials each of the three prepulse conditions and 12 pulse alone conditions presented in a fixed, pseudorandom order (Block 2). Then 5 more pulse alone stimuli (Block 3) were presented at the end of the paradigm for a total of 40 trials. Finally, EMG analysis was performed with Brain Vision Analyzer (Cortech Solutions, Wilmington, NC) after using a high-pass filter of 28 Hz at 12 dB/Oct. The waveform was then smoothed using a 40-Hz 24 dB/Oct low-pass filter. All trials were screened for errors. Startle data was wave-form averaged for each trial type within each block, baseline corrected and rectified. The highest point between 30 and 120 ms relative to baseline after onset of startle stimulus was defined as the peak startle response, or startle magnitude. Participants with a relative lack of startle response (<5 microvolts) to the second block of pulse alone stimuli were excluded from latency and PPI analyses per established methods (3). The following were examined: 1) reactivity, or the mean magnitude of startle response to pulse alone stimuli in blocks 1–3; 2) startle peak latency to the point of greatest magnitude for pulse alone and prepulse conditions in blocks 1 and 2; and 3) prepulse inhibition (PPI) in block 2. The latter measure was computed as a percentage [(startle magnitude to pulse alone − startle magnitude to prepulse + pulse conditions)/startle magnitude to pulse alone) × 100]. We previously established that the startle parameters are reliable between NAPLS sites (10).

### Statistics

SPSS version 26 (IBM, Armonk, NY) was used for statistical analyses. The analyses of reactivity, latency, and PPI were initially conducted assessing group, site, and demographic factors (e.g., sex and age) that are known to affect startle variables using repeated measures ANOVAs (3). Given the association between startle magnitude and peak startle latency (24), startle magnitude was used as a covariate in latency analyses. If assumption of sphericity was violated, Greenhouse-Geisser epsilon corrections were used to adjust degrees of freedom, and no results were altered. Follow-up analyses assessed effects of psychotropics, cannabis, and tobacco on startle measures given previous findings in the literature (3). Antipsychotic as well as stimulant treatment effects were analyzed in separate analyses in the CHR participants. To better understand all significant interaction effects (e.g., smoking, cannabis, group, sex, ISI, medication) on startle reactivity, latency or PPI, ANOVAs were used to deconstruct the omnibus analyses. Analyses to examine risk for psychosis in the CHR sample were then performed comparing CHR participants who developed a psychotic episode to those who did not within 1 year from the time of startle testing. Area under the receiver operating characteristic curve (AUC, equivalent to Harrell’s c-statistic) was used to test the ability of the model to correctly distinguish between psychotic outcomes (discrimination performance).

## Results

### Clinical Assessment and Demographics

The sample included 543 CHR and 218 NC participants (**Table 1**). There were significant differences in age but no group differences in sex distribution between NC and CHR participants. Therefore, age was included in analyses of experimental variables as a covariate. Given previous findings of sex differences in PPI (25, 26), sex was also used as a between subjects factor in analysis of startle measures. A greater percentage of CHR participants reported a history of tobacco and cannabis use. The CHR sample was more symptomatic per the SOPS and a greater percentage were prescribed stimulants and antipsychotics. Among the 543 CHR participants, 58 converted to psychosis within 1 year while 255 are known to have not converted to psychosis within this time period. Other CHR participants (N = 229, 42%) were either lost to follow-up or refused further participation prior to 1 year follow-up. There were no differences in age, sex distribution, cannabis use, tobacco use, antipsychotic use or stimulant use between those CHR participants who later converted to psychosis versus those who did not.

**Table 1:** Participant Characteristics.

	CHR (n = 543)	NC (n = 218)	Test Statistic t or Chi^2^	p
Male%	58.2	55.1	0.65	ns
Age (SD)	18.7 (4.4)	19.8 (4.9)	2.9	0.004
Tobacco%	23.3	10.0	20.0	0.001
Cannabis%	55.8	42.4	9.5	0.002
Stimulant%	6.0	0.5	11.1	0.001
Antipsychotic%	11.0	0.0	26.2	0.001
SOPS (SD)				
Positive	11.5 (4.0)	1.8 (3.0)	−35.5	0.001
Negative	11.4 (5.9)	2.3 (3.4)	−25.6	0.001
Disorganized	4.8 (3.1)	0.8 (1.4)	−23.1	0.001
General	9.0 (4.2)	1.3 (2.2)	−32.9	0.001

### Startle Magnitude

**Figure 1:**
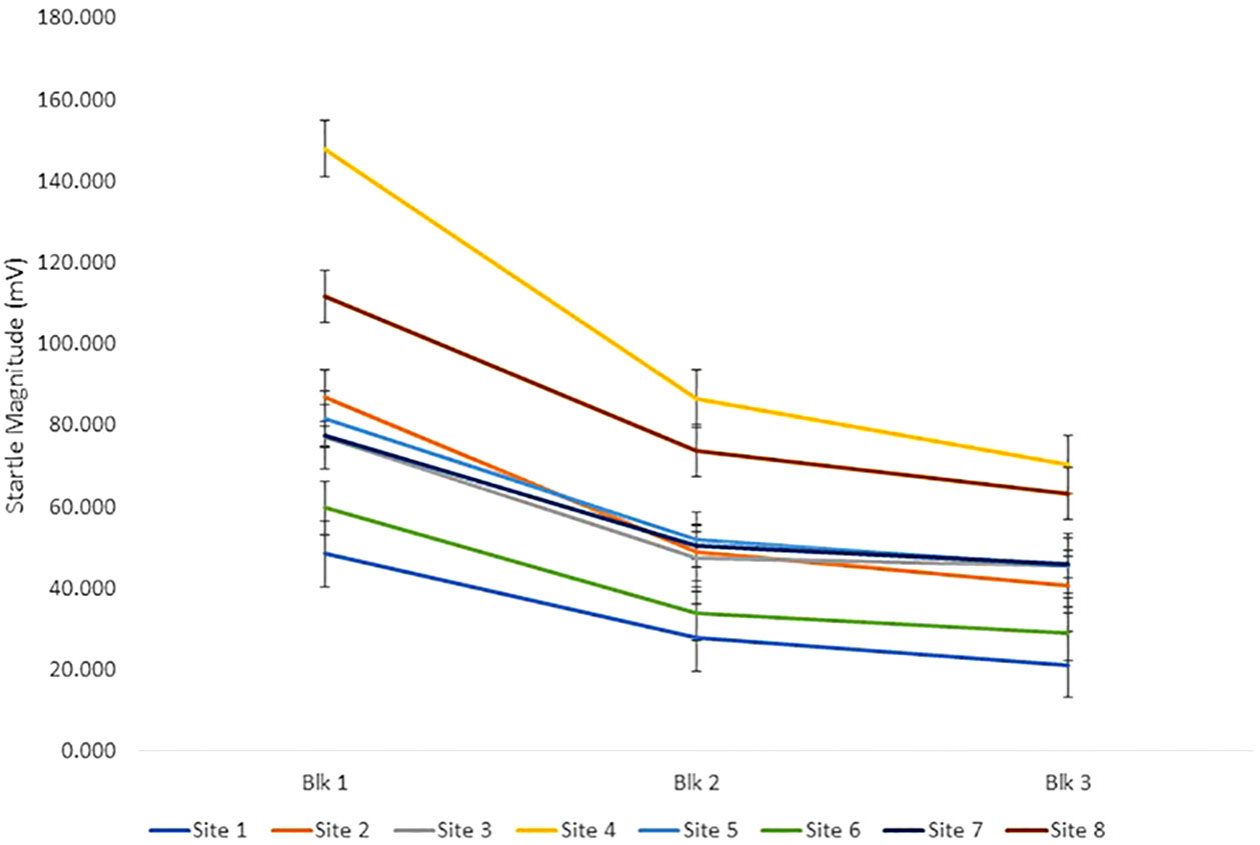
The figure shows startle magnitude to pulse alone stimuli across the three blocks of the startle session across all 8 sites. There were significant site effects despite equivalent equipment, calibration, and methods across sites.

Pulse alone trial reactivity was analyzed using a repeated measures ANOVA design with block (3 levels) as a within subject factor and group (2 levels—NC and CHR), sex and site as between subjects factors and age as a covariate. There were no significant age, group, or sex differences. There was a significant effect of block (F[2,8496] = 15.7, p<.001) due to habituation, or decrement in startle magnitude, across the session. There were also significant site (F[7,715] = 10.0, p<0.001) (**Figure 1**), site × block (F[14,715] = 8.9, p<0.001) and sex × block (F[2,715] = 5.7, p<0.005) effects. Females had greater startle magnitude in block 1 and less in block 3 accounting for this effect. The significant site and site × block effects are driven by site 4 having greater startle magnitude relative to all other sites and sites 1 and 6 having less startle magnitude relative to other sites (**Figure 1**). These site differences were most evident in block 1 when participants were first exposed to the loud, startling sounds. We have previously observed site differences in the same direction during a traveling subjects study that found good within and between site reliability but site differences in only startle magnitude (10). We have therefore reviewed all methodologic variables that might affect the stimuli or measurement including the equipment and settings, calibration, ambient noise, electrical noise, location of electrodes, instructions to subjects, and testing environment. To assure the loudness of the startle stimuli was accurate and consistent across sites, a decibel meter was mailed to other sites. No equipment or methodological differences were identified across sites (10).

#### Cannabis and Tobacco Effects on Startle Magnitude

When lifetime cannabis and tobacco use were each added as between subjects’ factors, there was no main or interaction effects due to these variables.

#### Psychotropic Medication Effects on Startle Magnitude

To assess medication effects, both antipsychotic and stimulant treatment were added as between subjects’ factors in analyses of CHR subjects. There were no significant main effects of antipsychotics or stimulants on startle reactivity within the CHR group.

#### Startle Magnitude Prediction of Psychosis

The baseline startle reactivity of 58 CHR participants who developed a psychotic illness within 1 year was compared to that of CHR participants (N = 255) who had not developed psychosis by 1 year follow-up and NC participants. There were no conversion main or interaction effects.

#### Startle Magnitude Summary

There were no group, age, psychotropic medication, tobacco, cannabis or conversion differences in startle reactivity or habituation. There were significant site differences in startle magnitude that are similar to those found in a previous traveling subjects study despite identical methods and equipment (10).

### Startle Latency

Startle latency to the first two blocks of startle pulse alone stimuli was analyzed using a repeated measures (block) ANOVA with group, sex and site as a between subjects’ factors with age and startle magnitude in block 2 as covariates. Startle magnitude was covaried given the correlation between startle magnitude and peak latency (24). There were no group, age, or site main effects but significant block (F[1,622] = 5.9, p<0.05) (block 1>block 2) and sex (F[1,622] = 6.7, p<0.01) (males > females) main effects as well as the expected significant startle magnitude effect (F[1,622] = 20.4, p<0.001) on startle latency and block × site × group interaction effects (F[7,622] = 2.2, p<0.05) (non-significant group differences that varied by block and site). In the prepulse latency conditions there were no significant main effects for group, age, site or sex but there were significant ISI effects (F[2,910] = 5.8, p<0.01).

#### Cannabis and Tobacco Effects on Startle Latency

**Figure 2:**
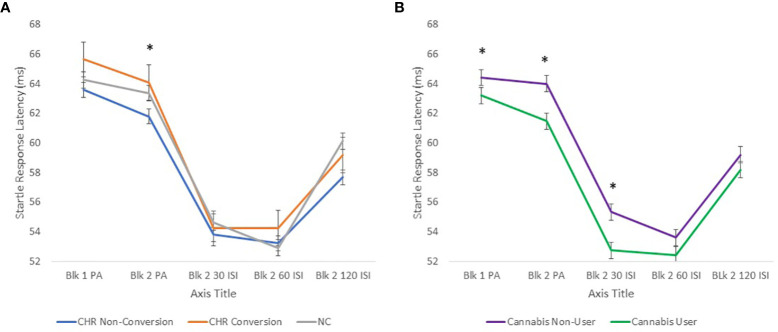
**(A)** shows startle latency to pulse alone and prepulse trials in CHR individuals who later converted to psychosis, those who did not convert to psychosis within 1 year and NC participants. CHR participants who later converted to psychosis had longer startle latency in the block 2 pulse alone condition compared to those who did not convert. **(B)** shows that Cannabis users had shorter startle latencies compared to non-users across all groups. *p<0.05.

When cannabis and tobacco use were added as between subjects’ factors, there was a significant cannabis effect in both the pulse alone (F[1,550] = 8.6, p<0.005) and the prepulse latency (F[1,550] = 5.8, p<0.05) conditions with those individuals with a history of cannabis use showing faster startle latencies relative to those who had never smoked cannabis (**Figure 2**). In addition, there was a significant sex × group × cannabis effect (F1,559] = 4.9, p<0.05) in the prepulse latency analysis. When males and females were analyzed separately, the significant cannabis (F[1,235] = 7.1, p<0.01) effects were only present in female participants accounting for the cannabis × group (F[1,235] = 6.4, p<0.05) effect. There were no main or interaction effects for tobacco in either the pulse alone or prepulse latency conditions.

#### Psychotropic Medication Effects on Startle Latency

There were no significant group effects of antipsychotics or stimulants on startle latency in CHR participants.

#### Prediction of Psychotic Risk and Startle Latency

**Figure 3:**
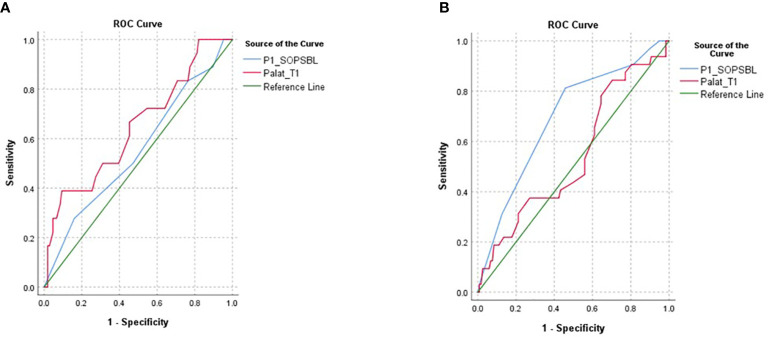
The figure shows AUC for female **(A)** and male **(B)** CHR participants since the significant conversion effects on startle latency were present in the females but not males. Startle latency (PA lat_t1) was more predictive of later conversion in females (AUC = 0.65) than males (AUC = 0.54) while P1, a measure of unusual thought content from the SIPS (P1_SOPSBL) was more predictive of later psychosis in males (AUC = 0.69) than in females (AUC = 0.55) but both were short of statistical significance.

When CHR participants who later converted to psychosis were compared to CHR participants who did not convert to psychosis within 1 year and NC participants, there was a trend group effect in the pulse alone trials (F[2,459] = 3.7, p = 0.056) due to longer startle latencies in CHR participants who later converted to psychosis versus those who did not convert to psychosis within 1 year (p<0.05, *post hoc* analysis) (**Figure 2**). There was also a significant Block × Sex × Conversion interaction effect (F[2,459] = 3.9, p<0.05) because the significant group differences were present in block 2 (F2,458] = 4.6, p<0.01, *post hoc* LCD conversion vs. non-conversion p<0.005) and present only in females (F[2,210] = 4.7, p<0.01). There were no significant main group or interaction effects in the prepulse latency conditions. AUCs were generated in females and males separately using block 2 pulse alone latency as well as P1 (unusual thought content) from the SOPS, a positive symptom domain found to predict conversion to psychosis in CHR participants and included in the NAPLS Psychosis Risk Calculator (27, 28). In male participants, P1 was more predictive of later conversion to psychosis (AUC 0.69) compared to startle latency (AUC 0.54) while the opposite was true in female participants where startle latency was more predictive (AUC 0.65) compared to P1 (AUC 0.55) (**Figure 3**). Comparisons of AUC between males and females for each of the variables showed trends that were short of significant (startle latency: z = −1.5, p = 0.1; P1: z = 1.9, p = 0.06).

#### Summary of Startle Latency Findings

There were no overall CHR vs. NC group effects in startle latency but the CHR participants who later converted to psychosis had significantly slower (i.e. greater) startle latency compared to those CHR participants who did not convert to psychosis within 1 year. This conversion group difference was most prominent in the second block of pulse alone stimuli and in females. In contrast, a history of cannabis use was associated with faster (i.e. shorter) latencies and this effect was driven primarily by female participants.

### Prepulse Inhibition

An omnibus ANOVA with ISI (3 levels: 30, 60, 120 ms) as a within subjects’ factor; group, site and sex as between subjects factors and age as a covariate was performed. No significant site or group effects were observed but there were significant ISI (F[2,1254] = 56.74, p<0.001, increased PPI with larger ISI), age (F[1,627] = 21.16, p<0.001; greater PPI with increasing age) and ISI × age (F[2,1254] = 5.23, p<0.01) effects and a trend group effect for sex (F[1,627] = 3.28, p<0.08; males had greater PPI than females).

#### Prepulse Inhibition and Age

**Figure 4:**
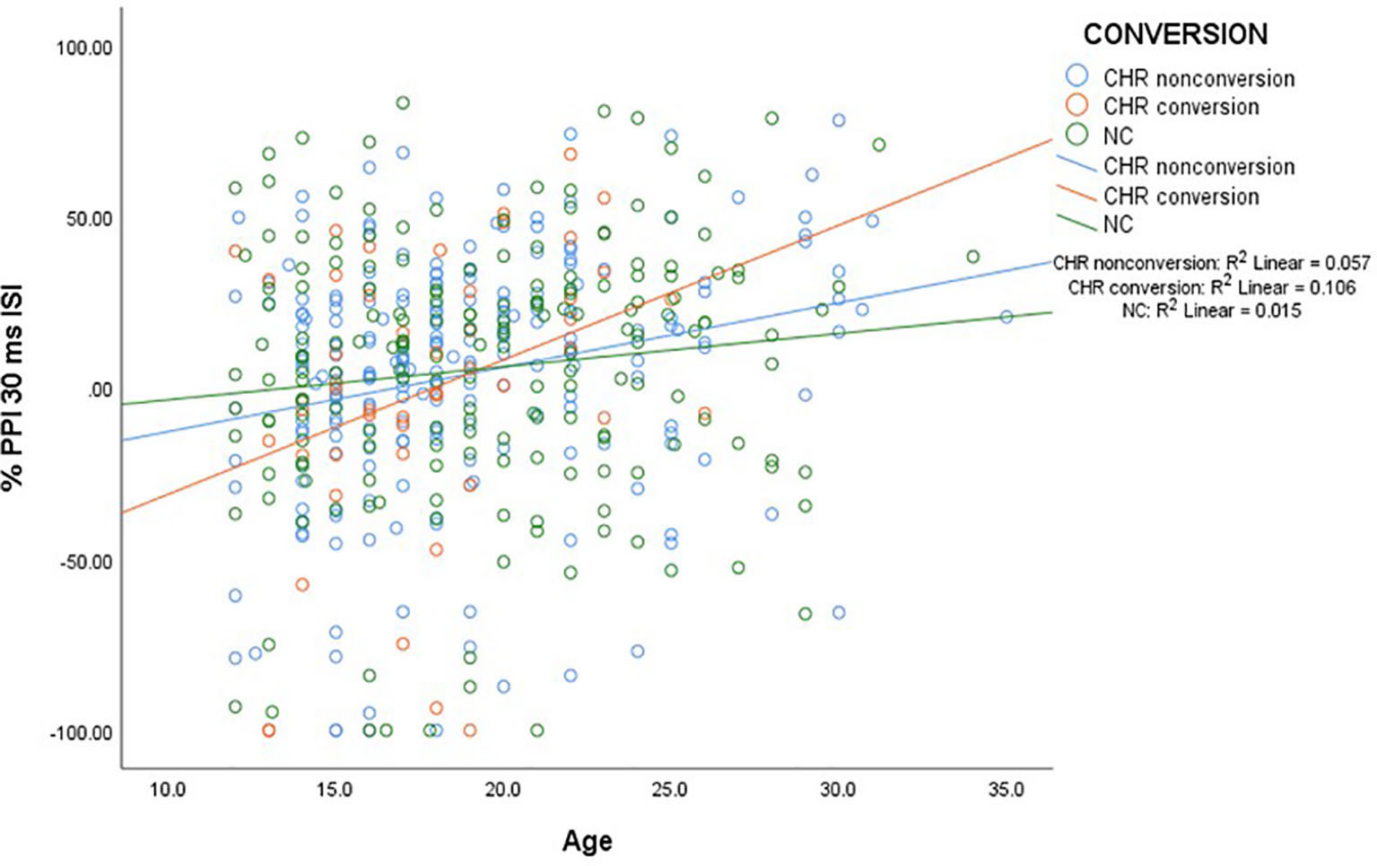
The figure shows a scatter plot of the significant age correlations with PPI in CHR converters, non-converters and NC participants. There were significant correlations within the CHR but not the NC participants. The CHR participants who converted to psychosis had the largest correlations with age.

To examine the significant age and age × ISI effects on PPI, Pearson Correlations between age and each of the three PPI conditions were performed in both CHR and NC participants. There were significant correlations with age in all PPI conditions but this was driven by the CHR and not the NC participants (**Table 2**). Interestingly, it was the CHR sample who later converted to psychosis who had the greatest correlations with age. **Figure 4** illustrates the correlations in the NC, CHR converters, and non-converters in the 30-ms ISI condition.

**Table 2:** Age was associated with PPI using Pearson correlations in participants from the NAPLS2 study.

	All Participants (N = 687)	Normal Comparison Participants (n = 197)	All CHR Participants (n = 490)	CHR Non-conversion, 1 year (n = 230)	CHR Conversion (n = 51)
30 ms ISI PPI × age	0.20**	0.07	0.26**	0.25**	0.28*
60 ms ISI PPI × age	0.16**	0.04	0.21**	0.25**	0.29*
120 ms ISI PPI × age	0.09*	0.04	0.12*	0.14*	0.22

When groups are separated, the significant correlations are evident only in the CHR groups but not the NC participants, with the largest correlations in those CHR participants who later converted to psychosis. *p < 0.05, **p < 0.01.

#### Cannabis and Tobacco Effects on Prepulse Inhibition

**Figure 5:**
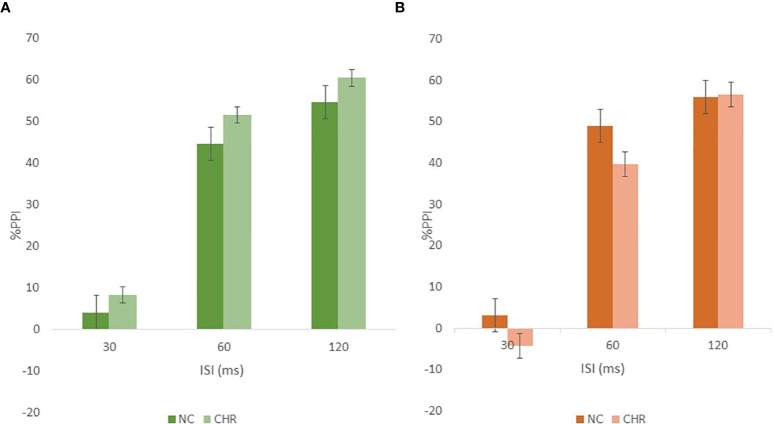
The figure shows the group by cannabis by PPI interaction in which cannabis users **(A)** had the greatest PPI and there were opposite group differences in cannabis users **(A)** versus non-users **(B)**.

The effects of cannabis and tobacco on PPI were analyzed using a repeated-measures ANOVA with cannabis use and tobacco smoking as between subjects factors in separate analyses. There were no main effects for cannabis use but the previously observed significant sex effect was no longer present in the cannabis analysis, and a significant cannabis × group interaction was present F[1,555] = 4.23, p<0.05), suggesting that sex differences in PPI may in part be accounted for by substance use in this sample as was previously observed by Cadenhead (3). The cannabis users and non-users were analyzed separately to further understand the cannabis × group interaction effect. There were no significant group or interaction effects in either the cannabis users or non-users but there were trend differences (CHR>NC in cannabis users and NC>CHR in non-users) in the opposite directions as shown in **Figure 5**. Similarly, when CHR and NC groups were analyzed separately, there were no significant cannabis main effects in the NC participants but in the CHR sample there was a trend toward cannabis users having greater PPI relative to cannabis non-users (F[1,403] = 3.55, p = 0.06). There were no significant main effects for tobacco but there was a significant sex × tobacco interaction (F[1,625] = 4.0, p<0.05) that was accounted for by a trend in the females (F[1,268] = 3.58, p = 0.06) having less PPI in smokers versus non-smokers while the male smokers and non-smokers did not differ.

#### Psychotropic Medication Effects on Startle PPI

There were no significant effects of antipsychotics or stimulants on startle PPI within the CHR participants.

#### Prepulse Inhibition and Risk of Future Psychosis

The baseline PPI data for CHR participants who later transitioned to psychosis was compared to that of CHR participants who had not developed psychosis by 1 year follow-up and NC. There were no significant conversion main or interaction effects.

#### Prepulse Inhibition Summary

In summary, the expected ISI effects on PPI were observed as well as a replication of an age effect we have observed previously (3) that was driven by greater PPI with increased age in the CHR sample that was greatest in those who later converted to psychosis. There was a trend toward sex effects and no group differences. Finally, there were no clear effects of psychotropic medication on PPI but there was a significant effect of cannabis on PPI in the CHR sample with cannabis smokers having greater PPI. Also, of interest is the fact that the normal sex effects on PPI (M>F) were no longer present when cannabis use history was added to the analysis.

## Discussion

This is one of the largest datasets of CHR participants with startle data who are known to have converted to psychosis after shorter term follow-up. Detailed analyses demonstrate evidence of group differences in startle measures linked to psychotic conversion and complex interactions between startle measures, age, sex, and exposure to cannabis and tobacco in early psychosis.

### Startle Reactivity

In this sample we did not observe significant group differences in startle magnitude or a relationship to tobacco, cannabis, or psychotropic medication. Previous animal and human literature has been mixed in regards to nicotine (29–37), antipsychotics (38, 39) and stimulant effects (40–42) on startle magnitude. Cadenhead (3) previously reported greater startle magnitude in an early psychosis sample who used cannabis but this finding was not observed in the current sample. The significant site effect for startle magnitude was found previously in a traveling subjects study (10). After extensive investigation, no methodological or equipment differences were identified that accounted for the site differences (10).

### Startle Latency

This is the first study to show greater startle latency in CHR participants who later convert to psychosis. This result is only present in females, in whom startle latency is more predictive of later psychosis than PI (unusual thought content) from the SOPS that is most predictive of psychosis in the CHR sample as a whole (27, 28). It is therefore possible that startle latency represents a biomarker of psychosis risk in female CHR participants but this finding clearly needs replication, ideally in a sample that is followed for greater than 1 year since up to 1/3 of future psychotic conversions occur after 2 years (43). Hasenkamp et al. (12) have reported longer startle latencies in patients with schizophrenia compared to NC participants. In addition, startle latency is highly heritable and linked to the neuregulin gene (NRG1) (44). Interestingly, female transgenic mice that overexpress NRG1 have more anxiety in behavioral tasks suggesting some sex differences related to expression of NRG1 (45). Startle latency is an index of neural processing speed. Slower latency is associated with worse cognitive performance (46), which is also associated with risk for psychotic conversion (47). The mechanism by which startle latency may be increased in schizophrenia and CHR participants who later convert to psychosis is unknown but substances such as nicotine have been shown to increase startle latency, most likely by causing dopamine release in the striatum (48). In the current study, tobacco use did not appear to be related to startle latency but increased dopamine release in CHR individuals on the verge of psychosis could account for this finding. Preclinical work indicates that latency is prolonged under conditions of increased dopamine stimulation (49, 50). In addition, a history of cannabis use was associated with shorter startle latencies in the current sample but most prominently in CHR males and NC females. It is unclear why cannabis users would have shorter latencies relative to non-users or by what mechanism this might occur.

### Prepulse Inhibition

Although we did not find group differences in PPI as noted in some (1, 2, 4, 5), but not all (3) CHR studies, we did find a significant age effect in CHR participants that Cadenhead (3) previously reported in a sample of CHR and first episode psychosis participants. In the current sample of teenagers and young adults, the significant age correlations were only present in the CHR sample but not NC participants. PPI is typically at a mature level by puberty (51), so the current replication of age effects in a CHR population suggests that the development of PPI is delayed in adolescents at risk for psychotic illness, consistent with neurodevelopmental abnormalities contributing to the emergence of psychosis. The positive correlations were greatest in those CHR participants who later converted to psychosis, suggesting evidence of neurodevelopmental immaturity or lag in the development of inhibitory circuitry in CHR participants. PPI age effects have been reported in older subject groups and show an inverse relationship with age (52, 53) in contrast to positive correlations noted in the young early psychosis population.

There was no evidence that PPI was associated with conversion to psychosis in the sample of 58 CHR participants who later converted to psychosis. The only other study to investigate conversion to psychosis was Cadenhead (3) who reported that a small sample of CHR participants (N = 6) who later converted to psychosis had greater PPI than both NC and CHR participants who did not convert to psychosis by 1 year. These findings are in contrast to the significant PPI deficits observed in patients with schizophrenia and their biological relatives (54).

Finally, there were no clear effects of psychotropic medication (55) or tobacco use on PPI as has been previously reported (56), but there was a significant effect of cannabis on PPI in the CHR sample with cannabis smokers having greater PPI. Also, of interest is the fact that the normal sex effects on PPI (M>F) were no longer present when cannabis use history was added to the analysis. There was also an interesting cannabis × group interaction due to opposite trends in cannabis users versus non-users. CHR participants who used cannabis had greater PPI relative to NC participants while non-users showed the opposite trend (>PPI in NC vs. CHR). Cadenhead (3) previously reported greater PPI in early psychosis participants who had a history of cannabis use and is in line with the finding of shorter starter latency being associated with cannabis use. The PPI paradigm has been used in translational studies of exogenous cannabinoids including cannabidiol (CBD) (57–59). Because many of the biochemical, physiological, and behavioral effects of CB1 receptor agonists can be reversed with CBD, recent studies have explored the possible role of cannabinoids in the treatment of psychosis (60). It is therefore possible that changes in the endocannabinoid system induced by cannabis use are reflected in PPI. Unfortunately the dosing or composition (THC to CBD ratio) of cannabis used was not controlled or assessed in this study although participants were not intoxicated at the time of startle testing.

### Caveats and Conclusions

Startle latency represents a potential biomarker of risk for psychosis but this finding needs to be replicated. PPI is likely sensitive to neurodevelopmental abnormalities given that it has now been found to be associated with age in early psychosis patients across two large studies (3) when no such association is evident in a NC sample. Finally, startle parameters (latency and PPI) are paradoxically faster (latency) and show greater inhibition (PPI) in participants who have a history of cannabis use, and this finding is most prominent in early psychosis and female participants. Given that the PPI finding is a replication, this suggests that there is still much to learn about the complex interaction between psychosis risk, sex, and epigenetic risk factors such as cannabis.

## Data Availability Statement

The raw data supporting the conclusions of this article will be made available by the authors, without undue reservation.

## Ethics Statement

The studies involving human participants were reviewed and approved by University of California San Diego, University of California Los Angeles, University of Calgary, Emory University, University of North Carolina Chapel Hill, Harvard University, Yale University, Northwell Long Island Jewish. Written informed consent to participate in this study was provided by the participants’ legal guardian/next of kin.

## Author Contributions

All authors were involved in developing the project, the analyses, drafting or reviewing the manuscript.

## Funding

This research was supported by a National Institutes of Health (NIH)-supported grants U01 MH081944 to KC; U01 MH066160 to SW, U01MH081984 to JA, U01 MH081902 to TC, UO1 MH081857 to BC, U01MH082004 to DP, U01 MH081928 to LS, and U01MH081988 to EW.

## Conflict of Interest

BC has been a consultant for Hoffman La Roche and received royalties for the CPT-IP. DP is on the Advisory Board for Sunovion DSMB, Genentech CNS, Genentech Mosaic Registry and a Consultant for Telesage. SW has been a Consultant for Merck. ED has received research support for work unrelated to this project from Auspex Pharmaceuticals, Inc. and Teva Pharmaceuticals, Inc. JJ was employed by Sage Therapeutics at the time of the study; Sage Therapeutics was not involved in the study design, collection, analysis, interpretation of data, the writing of this article or the decision to submit it for publication.

The remaining authors declare that the research was conducted in the absence of any commercial or financial relationships that could be construed as a potential conflict of interest.

## References

[1] De Koning MB Bloemen OJ Van Duin ED Booij J Abel KM De Haan L Pre-pulse inhibition and striatal dopamine in subjects at an ultra-high risk for psychosis. J Psychopharmacol (2014) 28(6):553–60. 10.1177/0269881113519507 24526133

[2] Togay B Cikrikcili U Bayraktaroglu Z Uslu A Noyan H Ucok A Lower prepulse inhibition in clinical high-risk groups but not in familial risk groups for psychosis compared with healthy controls. Early Interv Psychiatry (2020) 14(2):196–202. 10.1111/eip.12845 31264797

[3] Cadenhead KS Startle reactivity and prepulse inhibition in prodromal and early psychosis: effects of age, antipsychotics, tobacco and cannabis in a vulnerable population. Psychiatry Res (2011) 188(2):208–16. 10.1016/j.psychres.2011.04.011 PMC3114288 21555157

[4] Quednow BB Frommann I Berning J Kuhn KU Maier W Wagner M Impaired sensorimotor gating of the acoustic startle response in the prodrome of schizophrenia. Biol Psychiatry (2008) 64(9):766–73. 10.1016/j.biopsych.2008.04.019 18514166

[5] Ziermans T Schothorst P Magnee M van Engeland H Kemner C Reduced prepulse inhibition in adolescents at risk for psychosis: a 2-year follow-up study. J Psychiatry Neurosci (2011) 36(2):127–34. 10.1503/jpn.100063 PMC3044196 21266126

[6] Braff DL Grillon C Geyer MA Gating and habituation of the startle reflex in schizophrenic patients. Arch Gen Psychiatry (1992) 49(3):206–15. 10.1001/archpsyc.1992.01820030038005 1567275

[7] Cadenhead KS Carasso B Swerdlow NR Geyer MA Braff DL Prepulse inhibition and habituation of the startle response are stable neurobiological measures in a normal male population. Biol Psychiatry (1999) 45:360–4. 10.1016/S0006-3223(98)00294-7 10023514

[8] Abel K Waikar M Pedro B Hemsley D Geyer M Repeated testing of prepulse inhibition and habituation of the startle reflex: a study in healthy human controls. J Psychopharmacol (1998) 12(4):330–7. 10.1177/026988119801200402 10065906

[9] Ludewig K Geyer MA Etzensberger M Vollenweider FX Stability of the acoustic startle reflex, prepulse inhibition, and habituation in schizophrenia. Schizophr Res (2002) 55(1-2):129–37. 10.1016/S0920-9964(01)00198-0 11955972

[10] Cadenhead KS Addington J Cannon TD Cornblatt BA de la Fuente-Sandoval C Mathalon DH Between-site reliability of startle prepulse inhibition across two early psychosis consortia. Neuroreport (2013) 24(11):626–30. 10.1097/WNR.0b013e3283637845 PMC4135529 23799460

[11] Greenwood TA Braff DL Light GA Cadenhead KS Calkins ME Dobie DJ Initial heritability analyses of endophenotypic measures for schizophrenia: the consortium on the genetics of schizophrenia. Arch Gen Psychiatry (2007) 64(11):1242–50. 10.1001/archpsyc.64.11.1242 PMC10588564 17984393

[12] Hasenkamp W Epstein MP Green A Wilcox L Boshoven W Lewison B Heritability of acoustic startle magnitude, prepulse inhibition, and startle latency in schizophrenia and control families. Psychiatry Res (2009) 178(2):236–43. 10.1016/j.psychres.2009.11.012 PMC2902662 20483176

[13] Cadenhead KS Vulnerability markers in the schizophrenia spectrum: implications for phenomenology, genetics, and the identification of the schizophrenia prodrome. Psychiatr Clin North Am (2002) 25(4):837–53. 10.1016/S0193-953X(02)00021-7 12462863

[14] Gottesman II Gould TD The endophenotype concept in psychiatry: etymology and strategic intentions. Am J Psychiatry (2003) 160(4):636–45. 10.1176/appi.ajp.160.4.636 12668349

[15] Braff DL Freedman R The importance of endophenotypes in studies of the genetics of schizophrenia. Davis KLCD Coyle J Nemeroff C, editors. Baltimore, MD: Lippincott, Williams & Wilkins (2002). p. 703–16.

[16] Swerdlow NR Caine SB Braff DL Geyer MA The neural substrates of sensorimotor gating of the startle reflex: a review of recent findings and their implications. J Psychopharmacol (1992) 6(2):176–90. 10.1177/026988119200600210 22291349

[17] Swerdlow NR Braff DL Geyer MA Cross-species studies of sensorimotor gating of the startle reflex. Ann New York Acad Sci (1999) 877(2):202–16. 10.1111/j.1749-6632.1999.tb09269.x 10415651

[18] Geyer MA Krebs-Thomson K Braff DL Swerdlow NR Pharmacological studies of prepulse inhibition models of sensorimotor gating deficits in schizophrenia: a decade in review. Psychopharmacol (Berl) (2001) 156(2-3):117–54. 10.1007/s002130100811 11549216

[19] Powell SB Geyer MA Developmental markers of psychiatric disorders as identified by sensorimotor gating. Neurotox Res (2002) 4(5-6):489–502. 10.1080/10298420290030578 12754162

[20] Schneider M Koch M Chronic pubertal, but not adult chronic cannabinoid treatment impairs sensorimotor gating, recognition memory, and the performance in a progressive ratio task in adult rats. Neuropsychopharmacology (2003) 28(10):1760–9. 10.1038/sj.npp.1300225 12888772

[21] Addington J Cadenhead KS Cornblatt BA Mathalon DH McGlashan TH Perkins DO North American Prodrome Longitudinal Study (NAPLS 2): overview and recruitment. Schizophr Res (2012) 142(1-3):77–82. 10.1016/j.schres.2012.09.012 23043872 PMC3502644

[22] McGlashan T Walsh B Woods S The Psychosis-Risk Syndrome: Handbook for Diagnosis and Follow-Up. New York, NY: Oxford University Press (2010).

[23] Drake RE Rosenberg SD Mueser KT Assessing substance use disorder in persons with severe mental illness. New Dir Ment Health Serv (1996) 70:3–17. 10.1002/yd.23319960203 8754227

[24] Hoffman HS Searle JL Acoustic and temporal factors in the evocation of startle. J Acoust Soc Am (1968) 43(2):269–82. 10.1121/1.1910776 5636789

[25] Jovanovic T Szilagyi S Chakravorty S Fiallos AM Lewison BJ Parwani A Menstrual cycle phase effects on prepulse inhibition of acoustic startle. Psychophysiology (2004) 41(3):401–6. 10.1111/1469-8986.2004.00166.x 15102125

[26] Swerdlow NR Auerbach P Monroe SM Hartston H Geyer MA Braff DL Men are more inhibited than women by weak prepulses. Biol Psychiatry (1993) 34(4):253–60. 10.1016/0006-3223(93)90079-S 8399822

[27] Cannon TD Yu C Addington J Bearden CE Cadenhead KS Cornblatt BA An Individualized Risk Calculator for Research in Prodromal Psychosis. Am J Psychiatry (2016) 173(10):980–8. 10.1176/appi.ajp.2016.15070890 PMC5048498 27363508

[28] Carrion RE Cornblatt BA Burton CZ Tso IF Auther AM Adelsheim S Personalized Prediction of Psychosis: External Validation of the NAPLS-2 Psychosis Risk Calculator With the EDIPPP Project. Am J Psychiatry (2016) 173(10):989–96. 10.1176/appi.ajp.2016.15121565 PMC5048503 27363511

[29] Perkins KA Lerman C Coddington S Jetton C Karelitz JL Wilson A Gene and gene by sex associations with initial sensitivity to nicotine in nonsmokers. Behav Pharmacol (2008) 19(5-6):630–40. 10.1097/FBP.0b013e32830c3621 PMC2743299 18690117

[30] Duncan E Madonick S Chakravorty S Parwani A Szilagyi S Efferen T Effects of smoking on acoustic startle and prepulse inhibition in humans. Psychopharmacol (Berl) (2001) 156(2-3):266–72. 10.1007/s002130100719 11549228

[31] Grillon C Avenevoli S Daurignac E Merikangas KR Fear-potentiated startle to threat, and prepulse inhibition among young adult nonsmokers, abstinent smokers, and nonabstinent smokers. Biol Psychiatry (2007) 62(10):1155–61. 10.1016/j.biopsych.2006.12.027 PMC2111055 17543892

[32] Mueller V Mucha RF Pauli P Dependence on smoking and the acoustic startle response in healthy smokers. Pharmacol Biochem Behav (1998) 59(4):1031–8. 10.1016/S0091-3057(97)00508-X 9586864

[33] Della Casa V Hoefer I Weiner I Feldon J The effects of smoking on acoustic prepulse inhibition in healthy men and women. Psychopharmacology (1998) 137(4):362–8. 10.1007/s002130050631 9676896

[34] Robinson JD Cinciripini PM Tiffany ST Carter BL Lam CY Wetter DW Gender differences in affective response to acute nicotine administration and deprivation. Addict Behav (2007) 32(3):543–61. 10.1016/j.addbeh.2006.05.021 PMC4760691 16842931

[35] Acri JB Grunberg NE Morse DE Effects of nicotine on the acoustic startle reflex amplitude in rats. Psychopharmacol (Berl) (1991) 104(2):244–8. 10.1007/BF02244186 1876669

[36] Acri JB Brown KJ Saah MI Grunberg NE Strain and age differences in acoustic startle responses and effects of nicotine in rats. Pharmacol Biochem Behav (1995) 50(2):191–8. 10.1016/0091-3057(94)00285-Q 7740057

[37] Faraday MM O’Donoghue VA Grunberg NE Effects of nicotine and stress on startle amplitude and sensory gating depend on rat strain and sex. Pharmacol Biochem Behav (1999) 62(2):273–84. 10.1016/S0091-3057(98)00159-2 9972694

[38] Csomor PA Yee BK Feldon J Theodoridou A Studerus E Vollenweider FX Impaired prepulse inhibition and prepulse-elicited reactivity but intact reflex circuit excitability in unmedicated schizophrenia patients: a comparison with healthy subjects and medicated schizophrenia patients. Schizophr Bull (2009) 35(1):244–55. 10.1093/schbul/sbm146 PMC2643951 18245063

[39] Yee BK Russig H Feldon J Apomorphine-induced prepulse inhibition disruption is associated with a paradoxical enhancement of prepulse stimulus reactivity. Neuropsychopharmacology (2004) 29(2):240–8. 10.1038/sj.npp.1300323 14666120

[40] Kokkinidis L Anisman H Involvement of norepinephrine in startle arousal after acute and chronic d-amphetamine administration. Psychopharmacol (Berl) (1978) 59(3):285–92. 10.1007/BF00426636 104332

[41] Davis M Svensson TH Aghajanian GK Effects of d- and l-amphetamine on habituation and sensitization of the acoustic startle response in rats. Psychopharmacologia (1975) 43(1):1–11. 10.1007/BF00437607 1172255

[42] Bakshi VP Geyer MA Antagonism of phencyclidine-induced deficits in prepulse inhibition by the putative atypical antipsychotic olanzapine. Psychopharmacol (Berl) (1995) 122(2):198–201. 10.1007/BF02246096 8848537

[43] Powers AR Addington J Perkins DO Bearden CE Cadenhead KS Cannon TD Duration of the psychosis prodrome. Schizophr Res (2020) 216:443–9. 10.1016/j.schres.2019.10.051 PMC7539292 31806523

[44] Smith AK Jovanovic T Kilaru V Lori A Gensler L Lee SS A Gene-Based Analysis of Acoustic Startle Latency. Front Psychiatry (2017) 8:117. 10.3389/fpsyt.2017.00117 28729842 PMC5498475

[45] Deakin IH Law AJ Oliver PL Schwab MH Nave KA Harrison PJ Behavioural characterization of neuregulin 1 type I overexpressing transgenic mice. Neuroreport (2009) 20(17):1523–8. 10.1097/WNR.0b013e328330f6e7 PMC2880453 19829162

[46] Massa N Owens AV Harmon W Bhattacharya A Ivleva EI Keedy S Relationship of prolonged acoustic startle latency to diagnosis and biotype in the bipolar-schizophrenia network on intermediate phenotypes (B-SNIP) cohort. Schizophr Res (2019) 216:357–66. 10.1016/j.schres.2019.11.013 PMC7239737 31796306

[47] Seidman LJ Shapiro DI Stone WS Woodberry KA Ronzio A Cornblatt BA Association of neurocognition with transition to psychosis: baseline functioning in the second phase of the North American Prodrome Longitudinal Study. JAMA Psychiatry (2016) 73(12):1239–48. 10.1001/jamapsychiatry.2016.2479 PMC5511703 27806157

[48] Evinger C Basso MA Manning KA Sibony PA Pellegrini JJ Horn AK A role for the basal ganglia in nicotinic modulation of the blink reflex. Exp Brain Res (1993) 92(3):507–15. 10.1007/BF00229040 8454014

[49] Naudin B Canu S Costentin J Effects of various direct or indirect dopamine agonists on the latency of the acoustic startle response in rats. J Neural Transm Gen Sect (1990) 82(1):43–53. 10.1007/BF01244833 1976319

[50] Svensson L The role of the dopaminergic system in the modulation of the acoustic startle response in the rat. Eur J Pharmacol (1990) 175(1):107–11. 10.1016/0014-2999(90)90160-8 1969797

[51] Ornitz EM Startle modification in children and developmental effects. In: Michael E Dawson E Anne M Schell E editors. Startle modification: Implications for neuroscience, cognitive science, and clinical science. New York, NY, USA: Cambridge (1999). p. 245–66.

[52] Ellwanger J Geyer MA Braff DL The relationship of age to prepulse inhibition and habituation of the acoustic startle response. Biol Psychol (2003) 62(3):175–95. 10.1016/S0301-0511(02)00126-6 12633977

[53] Kumari V Aasen I Papadopoulos A Bojang F Poon L Halari R A comparison of prepulse inhibition in pre- and postmenopausal women and age-matched men. Neuropsychopharmacology (2008) 33(11):2610–8. 10.1038/sj.npp.1301670 18216776

[54] Cadenhead KS Swerdlow NR Shafer KM Diaz M Braff DL Modulation of the startle response and startle laterality in relatives of schizophrenic patients and in subjects with schizotypal personality disorder: evidence of inhibitory deficits. Am J Psychiatry (2000) 157(10):1660–8. 10.1176/appi.ajp.157.10.1660 11007721

[55] Fargotstein M Hasenkamp W Gross R Cuthbert B Green A Swails L The effect of antipsychotic medications on acoustic startle latency in schizophrenia. Schizophr Res (2018) 198:28–35. 10.1016/j.schres.2017.07.030 28732798

[56] Song L Chen X Chen M Tang Y Wang J Zhang M Differences in P50 and prepulse inhibition of the startle reflex between male smokers and non-smokers with first episode schizophrenia without medical treatment. Chin Med J (Engl) (2014) 127(9):1651–5. 10.3760/cma.j.issn.0366-6999.20133306 24791869

[57] Hlozek T Uttl L Kaderabek L Balikova M Lhotkova E Horsley RR Pharmacokinetic and behavioural profile of THC, CBD, and THC+CBD combination after pulmonary, oral, and subcutaneous administration in rats and confirmation of conversion *in vivo* of CBD to THC. Eur Neuropsychopharmacol (2017) 27(12):1223–37. 10.1016/j.euroneuro.2017.10.037 29129557

[58] Long LE Chesworth R Huang XF McGregor IS Arnold JC Karl T A behavioural comparison of acute and chronic Delta9-tetrahydrocannabinol and cannabidiol in C57BL/6JArc mice. Int J Neuropsychopharmacol (2010) 13(7):861–76. 10.1017/S1461145709990605 19785914

[59] Peres FF Diana MC Levin R Suiama MA Almeida V Vendramini AM Cannabidiol administered during peri-adolescence prevents behavioral abnormalities in an animal model of Schizophrenia. Front Pharmacol (2018) 9:901. 10.3389/fphar.2018.00901 30186164 PMC6113576

[60] Koethe D Giuffrida A Schreiber D Hellmich M Schultze-Lutter F Ruhrmann S Anandamide elevation in cerebrospinal fluid in initial prodromal states of psychosis. Br J Psychiatry (2009) 194(4):371–2. 10.1192/bjp.bp.108.053843 19336792

